# Attention directs actions in visual foraging

**DOI:** 10.1038/s41598-025-97986-1

**Published:** 2025-05-06

**Authors:** Jan Tünnermann, Anna Schubö

**Affiliations:** Cognitive Neuroscience of Perception and Action, Department of Psychology, Philipps-University Marburg, Gutenbergstraße 18, 35032 Marburg, Germany

**Keywords:** Visual foraging, Selective attention, Goal-directed actions, Selection for action, Naturalistic visual search, Neuroscience, Psychology

## Abstract

Visual foraging tasks, where participants collect items by touching or clicking on them, have become popular for investigating visual search. They probe selective attention in multi-target contexts through naturalistic goal-directed actions, unlike the button presses used in many other paradigms. Despite their potential, such tasks had not been used to examine the interplay of attention and goal-directed actions until now, even though this topic has been extensively studied with other paradigms and has significant implications for understanding human visual behavior in the real world. In this study, we applied the visual foraging paradigm to address this gap. We found that attentional prioritization of one part in a two-part compound object is accompanied by a motor bias in the collecting action (stylus tap) toward the prioritized part. This bias combines with motor precision demands, such as aiming for stable contact points. Our findings show that action planning not only modulates the attentional landscape at large but also that attentional asymmetries (e.g., prioritizing one object part) feed back into the motor system, combining with motoric factors to refine goal-directed actions.

## Introduction

Planning and execution of goal-directed actions are closely tied to selective visual attention. While this connection is very evident in eye movements, which are usually preceded by attention shifts^1–4^, it also extends to manual reaching and grasping^5–9^. When it comes to motor planning, attention may serve two primary functions: selecting the spatial goals of the movement and prioritizing the relevant visual features for the action^10,11^. Some theories even suggest that the early information selected by attention can be directly fed into motor control to guide target-directed actions in certain situations, bypassing the need for perceptual-level representations^12–15^.

But the interplay between action and attention is not unidirectional: Research has also demonstrated that action intentions and affordances can influence attentional selection^16–19^, and more generally it has been suggested that both perception and action planning operate on the same neural codes^20–22^ and interact continuously during the processing of information^23,24^. Given the close relationship of attention and action it seems imperative that the two should be studied in conjunction. However, selective attention is typically investigated in search tasks in which simple button presses instead of goal-directed actions operationalize the response. When goal directed actions are included^6,25^, the experiments often consist of briefly shown discrete trials, whereas humans typically show continuous behavior when interacting with their environment.

Over the last ten years, the visual foraging paradigm, a computer-based experimental task in which participants search for and collect items within “patches”, has gained popularity in the study of selective attention^26–29^. The foraging task assesses visual search in more naturalistic scenarios with multiple targets, continuous interaction with the search environment, and directed actions toward the selected items. For instance, foragers collect items via mouse clicks^30^, tablet-stylus taps^29^, finger touches^26^, or virtual reality input devices^31^ (see also Ref. [32]) Especially the requirement to perform natural, goal-directed actions makes the task highly interesting for studying the interplay between action and attention. The search through patches requires a fast succession of selection actions performed toward the targets (often with multiple selections per second), putting high demands on efficient attention guidance and motor control. Many variants of the task do not require further decisions about the target at post-selection stages^33^; rather, it seems plausible that targets can already be collected after attentional selection. For instance, when picking raspberries from a bush, prioritizing the color of ripe berries typically leads to high-quality selections without the need for closer inspection—unless, of course, one is concerned about the occasional worm. Target templates of sought-for objects are stored in visual working memory^34,35^, and depending on their complexity, switching between them might be costly. However, when no switches are required or templates only contain a single feature, foraging is highly efficient (in so called “cruise phases”^28^), and foragers seem to engage in rapid attend–fixate–act cycles, in which actions could be directly targeted at the attended locations.

It has been repeatedly demonstrated that foragers tend to select targets of the same type in runs when the targets are defined by conjunctions (“conjunction foraging”). For instance, when green squares and red circles are targets and red squares and green circles are “distractors” (objects to be ignored), foragers collect items of the same type (e.g., green squares) for extended periods and then switch to the other type (e.g., red circles). This effect is often so extreme that some foragers collect all items of one type before switching to the second type^26,31,36,37^, and it may be a basic strategy to avoid target switching costs during search, which has also been observed in animals such as chickens^38^.

The studies discussed above highlight that efficiently switching and maintaining target templates are crucial for foraging performance, and that foragers adjust templates to reduce their load. For single-target search it has been shown that attention guidance does not necessarily work on highly precise, complete representations of the target. Instead it uses “good enough” target representations that allow fast guidance leading to mostly successful selections, limiting target templates to sufficiently diagnostic features or object parts^39^. Even though there is no direct evidence from visual foraging up to now, it seems very likely that good enough guidance is employed during foraging to minimize attentional load.

**Fig. 1:**
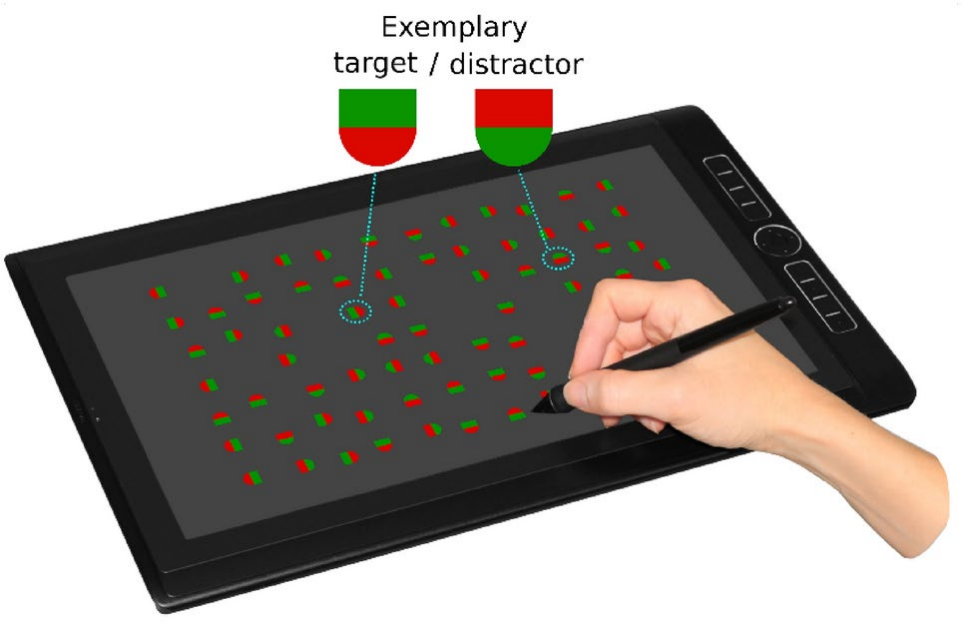
Tablet-PC with stimuli to scale. In the example, eight targets have already been collected. One exemplary target and one distractor are shown in magnification. Exemplary photo of the interaction with the tablet-PC, adapted from Ref. [40] / CC-BY 4.0.

In the present study, we investigated whether attention not only selects the target objects (and allows to discard non-targets) but also feeds local priority information into action control in order to refine the collecting actions. We combined the ideas of conjunction foraging and good-enough attention guidance, and constructed a new stimulus type, “conjunction objects”. A conjunction object has two distinct but spatially adjacent parts. For instance, half of a target is a red half-circle, and the other half is a green half-square. Distractors then consist of green half-circles and red half-squares (see Fig. 1). If foragers use “good-enough” guidance, they will not represent both parts of a target in a template but restrict the template to one of the parts (e.g., red half-circle), given that such a partial template is sufficient to find all targets. Moreover, as foragers are highly reluctant to switch between templates that require feature conjunctions, they will continue to search for the same partial target templates over extended periods in runs. Therefore, if attentional mechanisms not only control target selection but also direct the manual collecting actions, these actions should be biased toward the prioritized part, increasing the likelihood of the same part being repeatedly tapped when collecting items. Even though the resulting object-part runs are likely shorter than runs in traditional conjunction foraging (due to motor noise and other motoric influences), we expect them to provide evidence for a direct influence of attentional priority on the execution of the collecting action.

## Method

### Participants

We recruited 20 participants (10 female, 8 male, 2 diverse) for the experiment. All were right-handed (self-report). The sample size was decided with a simulation-based power analysis, which revealed a power approaching 1 (lower HDI bound 0.97) for detecting sub-random switching (switching probability < 0.5) in tapping on the object parts under conservative assumptions. Further details about the assumptions (e.g. assumed template switching probability, assumed motor noise, simulation algorithm, etc.) can be found in the Supplementary Information. Participant ages ranged from 19 to 33 years (*M* = 24.6, *SD* = 4.48), and all participants had normal or corrected to normal visual acuity and color vision (tested with an Oculus Binoptometer3). Participants provided informed consent, the experiment adhered to the 1964 Declaration of Helsinki, and the study was approved by the Ethics Committee of the Faculty of Psychology at Philipps-University Marburg.

### Apparatus and stimuli

The patches were presented on a large (345 × 195 mm) tablet-PC (WACOM MobileStudio Pro 16) with a resolution of 1920 × 1080 pixels, running at 60 Hz (see Fig. 1). At a typical viewing distance of 65 cm the display extended over 29.7° ×  17° of visual angle. All measurements in degrees below refer to degrees of visual angle at this distance. Participants interacted with the experiment using the tablet stylus. The experiment was implemented in OpenSesame^41^ using the PsychoPy^42^ backend.

Stimuli were “conjunction objects” composed of red and green half-circles (radius 23° pixels, 0.37°) and rectangles (46 × 236 pixels, 0.73° × 0.37°), see insets in Fig. 1. Targets consisted of green rectangles and red half-circles while distractors had red rectangles and green half-circles; this color–shape mapping was inverted for half of the participants. The green color was implemented with RGB = (0, 150, 0) and the red color with RGB = (207, 0, 0) matched in luminance (as close as possible on the device: red 28.7 cd/m2, green 28.2 cd/m2). The objects were positioned randomly on a 12 × 7 grid centered on the screen, extending over 1430 × 780 pixels (22.36° × 12.37°). A random jitter of 0 to ± 20 pixels (± 0.32°) was added to the *x*- and *y*-coordinates. Moreover, the objects were randomly rotated into all four cardinal orientations.

### Procedure

After receiving the instructions, participants performed one practice trial (unrecorded). Subsequently, they worked through 20 experimental trials, during which they collected 42 targets among 42 distractors per patch (trial). Each correct target contributed one point to their score, while any collected distractor incurred a penalty of minus five points (and an auditory beep). The trial ended when all targets in the patch were collected, and a point-based performance feedback was shown. This feedback included the earned positive points, the deducted negative points, and a “speed bonus” calculated as max(30 − *t*, 0) points, with *t* denoting the time taken to complete the patch in seconds. Additionally, participants were provided with an overview of their overall score in the current trial, as well as the accumulated score in the experiment so far. It was the goal to maximize the point score by working fast and accurately. Participants were further instructed to use the tablet stylus like a normal pen; the tablet ignored touches with the heel of the hand.

**Fig. 2:**
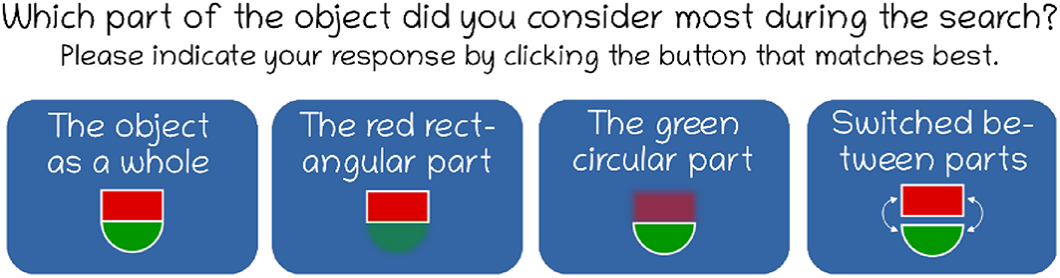
Question and response options in the search template self-report after the foraging task. Whether the “red rectangle / green half-circle” or “green rectangle / red half-circle” elements were shown depended on which of the two versions the participants were instructed to search. Note that the appearance in the figure was slightly altered for better presentation here: Texts were translated from German to English, font size was increased, and the gray background color was replaced with white.

After the foraging task, participants indicated which features they believed had guided their search, pressing one of four buttons that contained a text and an icon representation of a guiding template, containing the whole object, only the rectangular part, only the circular part, or switching between both parts (see Fig. 2).

### Data analysis

#### Statistical framework

As a statistical framework we employed Bayesian parameter estimation and report the mode (most likely value) followed by the 95% highest-density interval (HDI) in square brackets^43^. Models were implemented with PyMC^44^; samples were obtained using NUTS^45^ and assessed and visualized with ArviZ^46^ and Seaborn^47^.

#### End peak factor

Inter-target times (ITTs) refer to the time between successive target sections. Typically, ITTs rise toward the end of exhaustive foraging trials when participants search for last target. The strength of the rise is related to search difficulty. To quantify how much higher the last ITT in a trial was compared to a typical ITT from a “cruise phase” (the phase when many targets are available and foragers proceed swiftly^31^), we divided each trial’s last ITT by the median ITT and averaged this “end peak factor” across trials for every participant. The resulting average end peak factors were then submitted to Kruschke’s Bayesian version of the *t* test^48^.

#### Switching probability

The probability with which observers switched between tapping one or the other object part, our main variable of interest, was estimated from the count data (number of switches out of the switching possibilities within a trial) with a Bayesian hierarchical beta-binomial model^49^ which was also applied to foraging data in earlier studies^29,30^. The model estimates the group-level switching probability ${p}_{\mu }^{\text{switch}}$ and each participant $i$’s switching probability ${p}_{i}^{\text{switch}}$. The priors were chosen to be flat over the entire range (0 to 1) of possible probabilities. The correlation between switching in the first and second half of the experiment was modeled with a multivariate Normal (on the log-odds scale) on the group-level. See Supplementary Information for further details on the model specifications and priors. Note that in contrast to earlier studies that looked at switching between object types presented at different locations, we calculate switching probability for spatially adjacent object parts. Hence, switching probability is also affected by motor performance and can reveal whether attentional biases translate into a motoric ones.

#### Object part preference

To estimate the general preference of attending to one object part over the other, we obtained the count of “red object part tapped” (“red” being an arbitrary choice here) out of all collected targets in a trial and estimated the probability of selecting the red part with a similar model as described above. The difference of this model and the one described above is that here we use a non-hierarchical version, because the individual attentional preferences (in favor of certain colors of shapes) are probably highly individual therefore no common group tendency is modeled. Model details can also be found in the Supplementary Information.

## Results

**Fig. 3:**
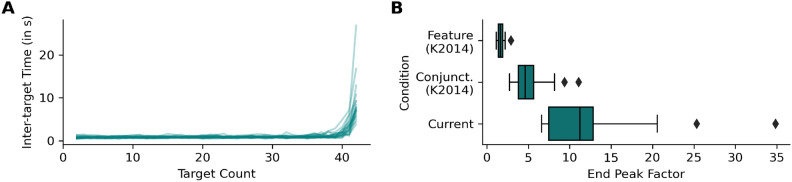
(**A**) Average (over trials) inter-target times at different target counts (number of collected items) in the current study. One line per participant. (**B**) Average end peak factors (last ITT divided by median ITT) for the current study and Kristjánsson et al.’s 2014 study^26^. Boxes delimit the interquartile range (IQR). Vertical bars inside the boxes represent the median. Whiskers extend to include the lowest/highest data points in a range from the first quartile minus 1.5 times the IQR to the third quartile plus 1.5 times the IQR. Points outside the range are plotted explicitly.

Participants took on average 49.28 (*SD* = 11.71) seconds to complete a patch and the error rate (taps on distractors) was generally low (*M* = 1.25%, *SD* = 1.34). As the current study is hinged on the idea that a difficult search presses participants into run-like foraging behavior, we first turn to performance measures based on the inter-target time (ITT, time between successive target taps) that can reveal if this was successful. The median ITT was on average 702.33 ms (*SD* = 118.66), and ITTs strongly increased toward the end of trials (ITT of the last tap: *M* = 8788.57 ms, *SD* = 5193.41, see Fig. 3A).

Pronounced “end peaks” in the ITTs are commonly found in conjunction foraging tasks, reflecting increased search difficulty^31^. As ITTs can depend on many factors (e.g. layout of the items or salience of the targets) the absolute height of the end peaks is difficult to compare between studies. To facilitate a comparison nevertheless, we calculated “end peak factors” that quantify the increase in ITT at the trial end relative to earlier ITTs (see Data Analysis). We compared the end peak factor to feature and conjunction foraging data from Kristjánsson et al.’s 2014 study^26^. A descriptive representation of the end peak factors can be seen in Fig. 3B, which shows that the end peaks in the current study are about 10 times (and for some participants up to 35 times) as high as the earlier ITTs in the trial. This does not only exceed typical end peaks in feature foraging but also the already high end peaks in typical conjunction foraging (see first and second row in Fig. 3B). Statistically, the end peak factor of the current study is 6 units [3.6 to 8.6]^HDI95%^ (or 2.3 times [1.6 to 3]^HDI95%^) higher than the end peak factor we calculated for the conjunction foraging data from Kristjánsson et al.’s 2014 study^26^, with zero (no difference) far outside the 95% HDI. Note that we did not test for mid-peaks^28^, which can be indicative for switches between runs in conjunction foraging. However, they only occur if switching between very long runs aligns near the center of the collection sequence. This is unlikely to occur in the present experiment because switching is not strictly necessary (participants could stick with one object part), and “random” switches can occur everywhere due to motor noise.

### Switching probability

**Fig. 4:**
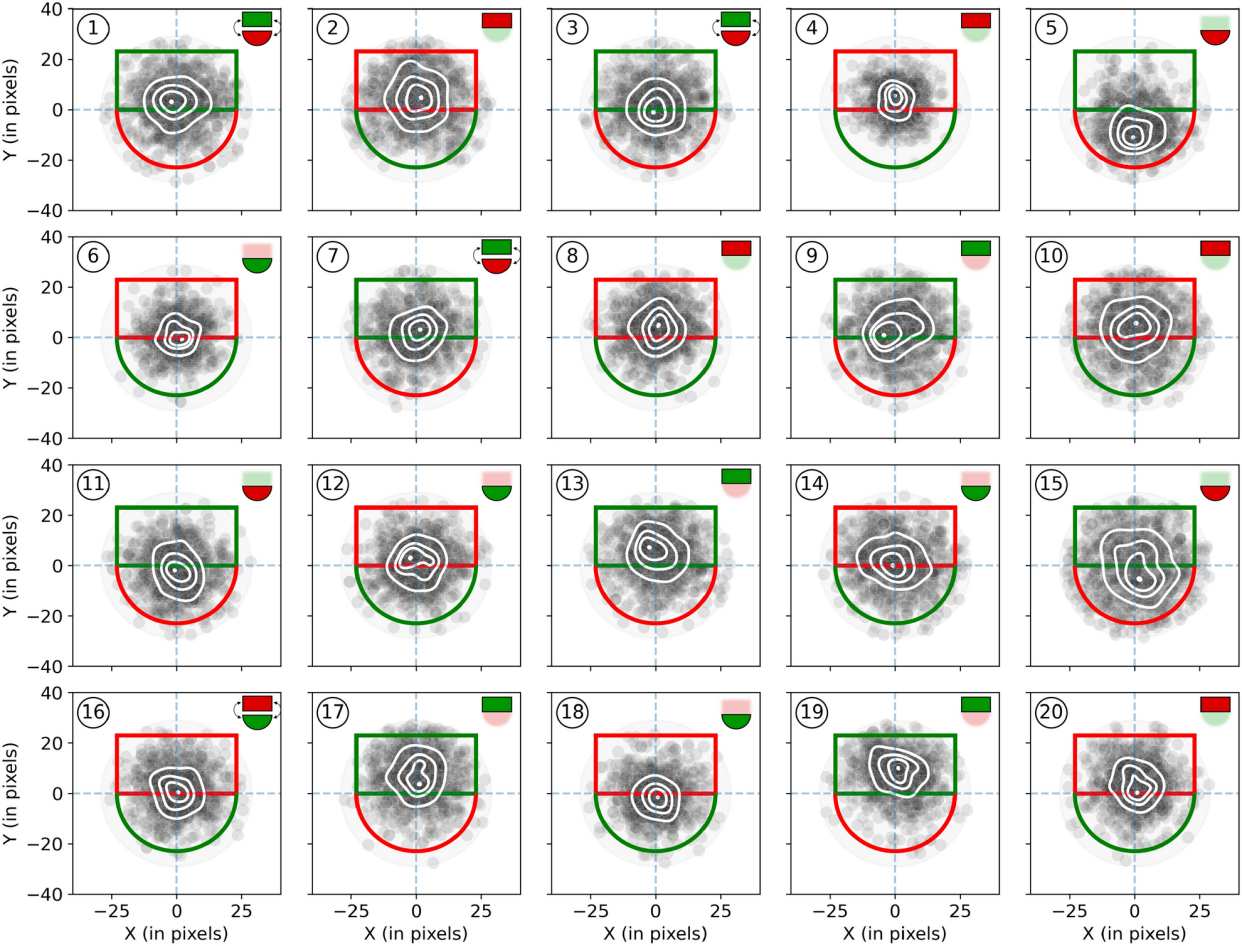
Tap position plots for all twenty participants. The rectangles and half-circles represent the targets (normalized to the same upright orientation) and their parts with their different colors. The small gray semi-transparent dots show the individual taps from all selections from all trials. The white markings were generated with Gaussian kernel density estimation. The white dot indicates the highest density the increasingly larger contours indicate levels that contain 10%, 25%, and 50% highest density. The faint gray circular area in the background represents the radius from the object center within which taps were accepted to collect the object. The little icons in the upper right corners represent the self-reported template use by highlighting the reported part(s). Figure best viewed in color.

Figure 4 shows “click maps” for each participant that indicate where observers tapped on the object. Note that this data has been normalized by rotating the coordinates into an upright orientation for stimuli that were presented in other orientations. These plots already indicate that participants have not switched randomly between the two object parts but often preferred attending to one part over the other (most strikingly seen for participants 4, 5, 17, and 19).

**Fig. 5:**
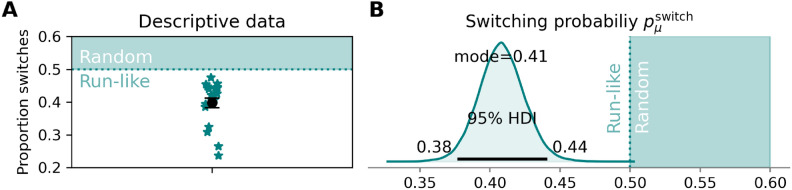
(**A**) Descriptive plot of the proportion of switches between the object parts produced by each participant (stars) when foraging. The black dot with error bars indicates the average and standard error of the mean, both calculated on arcsine-square-root-transformed data and transformed back to a proportion scale. (**B**) The posterior distribution of the switching probability estimated for the group level. In both (**A**) and (**B**) the white background area indicates run-like behavior (repeated selections of the same object part beyond those that happen randomly) and the turquoise area indicates random switching (note that technically only the probability of 0.5 indicates truly random switching, while probabilities larger than 0.5 indicate increasingly systematic alternations between the alternatives, typically not seen in visual foraging data).

Figure 5 shows the probabilities with which the participants switched between the object parts, the main variable of interest in the current study. As can be seen descriptively in Fig. 5A, the average proportion of switching of all participants is below 0.5, the level which would indicate entirely random (flexible) switching. Figure 5B shows the estimated switching probability on the group level ${p}_{\mu }^{\text{switch}}$ from the model-based analysis (see Data Analysis). This statistical analysis locates the central tendency of the switching probability at 0.41 [0.38, 0.44]^HDI95%^, with 0.5 (random switching) comfortably outside of the 95% HDI.

**Fig. 6:**
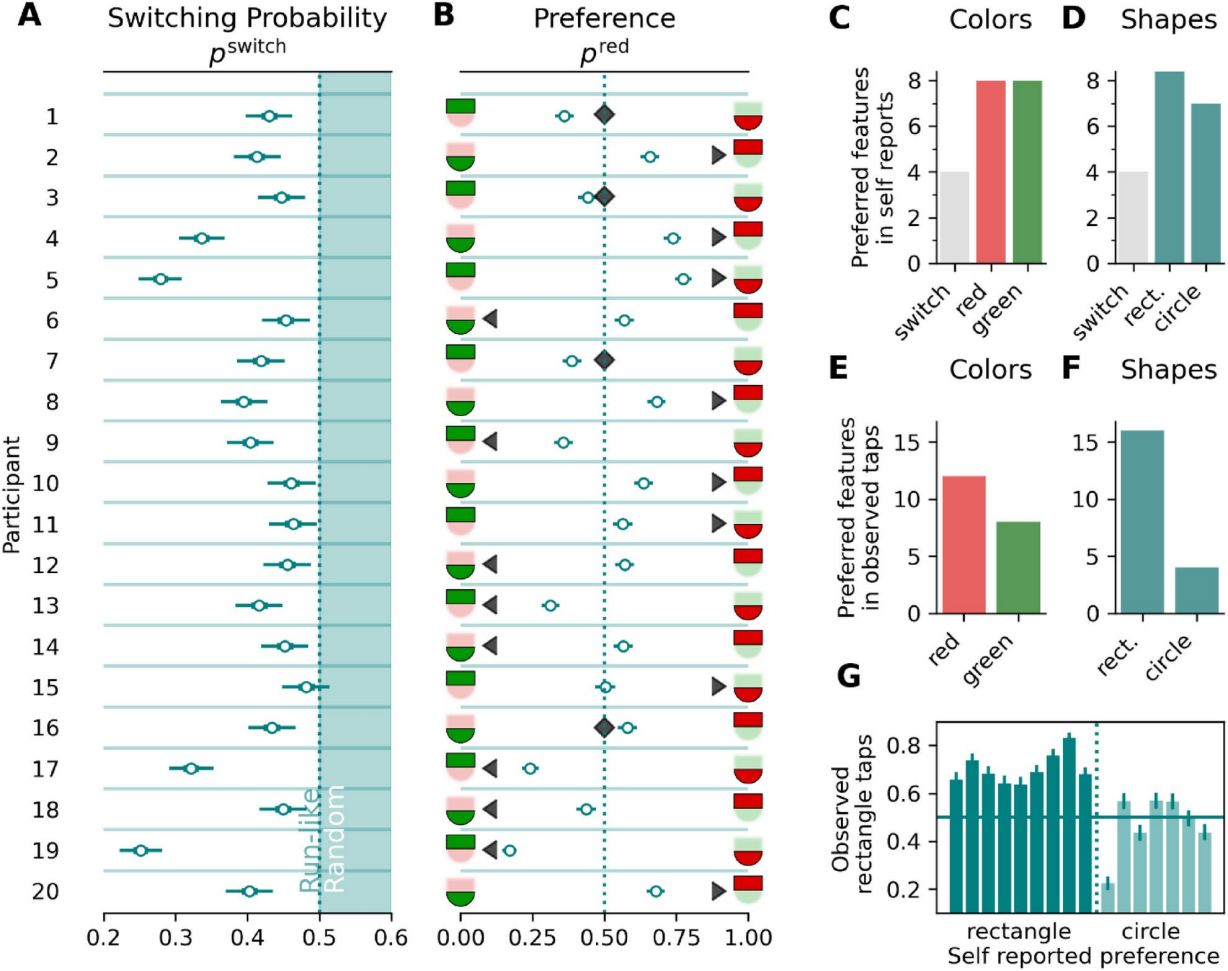
(**A**) Posterior of the switching probability ${p}^{\text{switch}}$. The white area in the panel represents run-like selections of the object parts and turquoise shaded area random switching (and systematic alternating, see note in Fig. 5). (**B**) Circle markers show posterior means of the preference for tapping on red parts (${p}^{\text{red}}$) on the participant level. Horizontal bars the 95% highest-density intervals. The triangular markers point to the template icon representing the self-reported preference. Diamond markers indicate participants who reported switching between parts. Panels (**C**) and (**D**) show the counts with which object parts in a certain color and shape were reported in the self-reports across participants (cf. triangle markers in panel **B**). Panels (**E**) and (**F**) show the counts of participants who preferred tapping one or the other feature (cf. circle markers in panel **B**, relative to 0.5). Panel (**G**) visualizes the proportion (model estimates) of rectangle tapping preferences for participants who reported having attended to the rectangle and those who reported having attended to the circular part. Each bar is one participant (four participants who reported switching were excluded), the error bars represent the 95% HDIs.

Figure 6A illustrates the participant-level estimates for object part switching probabilities. It is evident that all of these estimates fall below the random switching probability of 0.5, with the vast majority having 95% HDIs not overlapping with 0.5. Only for participant 15 the 95% HDI overlaps with 0.5. While the majority of participants tend to cluster between 0.4 and 0.5, a few exhibit lower switching probabilities, with some as low as 0.3 or even 0.25.

### Object part preference

In contrast to the switching probability discussed above, the preference for tapping on one part or the other was examined using a non-hierarchical model, which accommodates entirely independent preferences, because there might be strong individual differences. The resulting tapping preference estimates are illustrated in Fig. 6B, showing the probability of reporting the red object part.

To ascertain whether these individual preferences arise from attentional selection or stem from attention-independent biases towards specific features, we examined the frequency of reported features. Figures 6C and D illustrate the counts of feature values from the *self-reported* template, which are relatively balanced, with only the rectangle being reported marginally more often than the circle. Figures 6E and F depict the *observed* tapping preferences as counts (of the most commonly selected features of each participant), revealing a notably greater imbalance compared to the self-reported features, indicating a more pronounced preference for the rectangle. Figure 6G illustrates the model-estimated proportions of rectangle tapping preferences among participants who reported attending to the rectangle and those who reported attending to the circular part. The data reveal a correspondence between subjective reports and objective behavior. While some participants who reported attending to the circular part made more rectangle taps objectively, the frequency of such taps is substantially lower compared to participants who reported attending to the rectangle.

**Fig. 7:**
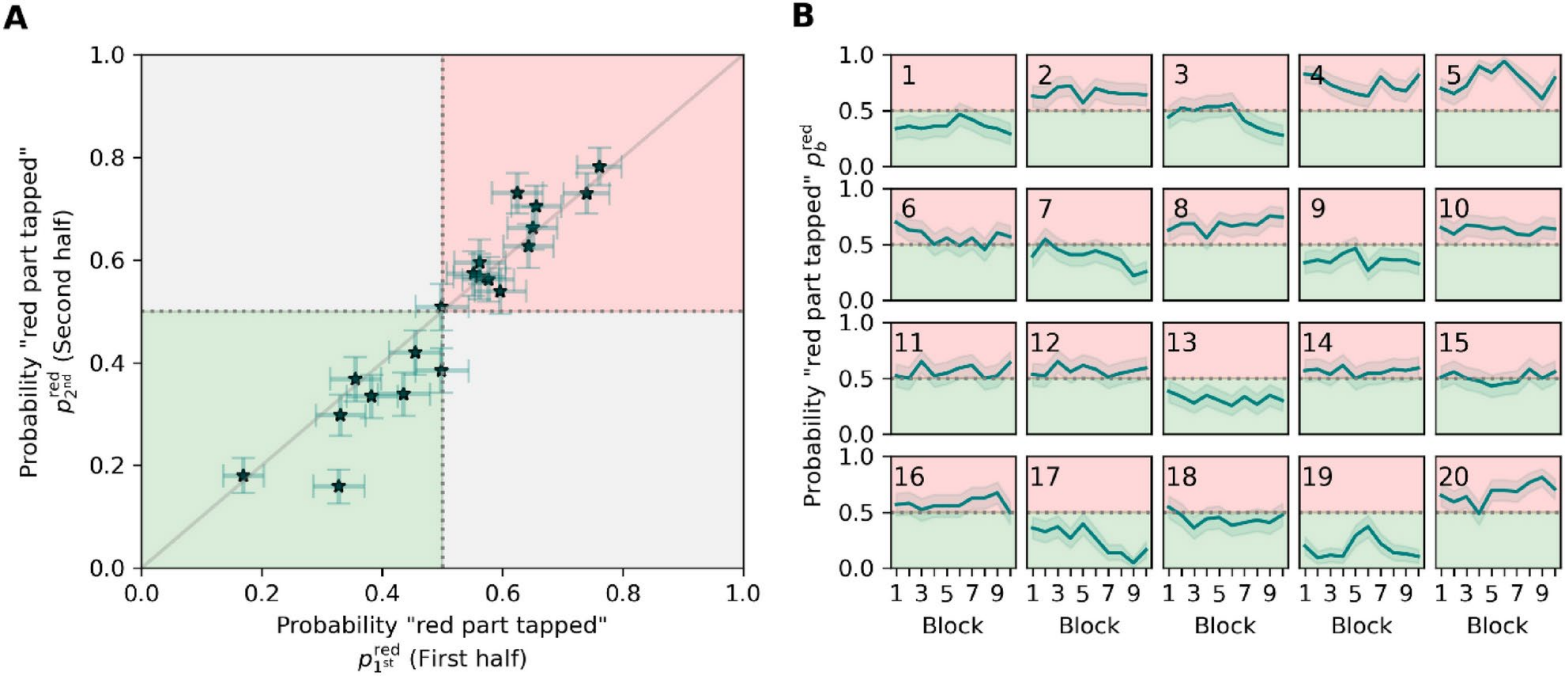
(**A**) Correlation between the tapping preferences for red parts estimated from the first (${p}_{\text{1st}}^{\text{red}}$) and the second (${p}_{\text{2nd}}^{\text{red}}$) half of the experiment. Points indicate the marginal posterior modes and error bars the 95% highest-density intervals. The green shaded area contains the values that correspond to preferring the green object part in both experiment halves and the red shaded area shows consistent preference for the red part. (**B**) Estimated tapping preferences of the red object parts (${p}_{b}^{\text{red}}$) over ten successive blocks $b$ of the experiment. The color of shaded areas indicates color of preference. Lines depict the posterior modes and the faint error bands the 95% highest-density intervals.

We further evaluated the consistency of object-part tapping preference over time by estimating the correlation between preferences in the first and second halves of the experiment (see Fig. 7A). The correlation coefficient, estimated at 0.88 [0.75, 0.98]^HDI95%^, attests to a robust correlation. This stability in tapping preference is further corroborated by the time-course analysis depicted in Fig. 7B, demonstrating a sustained preference of the same object part throughout the experiment. Additionally, no systematic trend is evident, suggesting that preferences did not consistently intensify or diminish over the course of the experiment.

### Template strategy (self-reported)

The striking outcome of the post-experiment question about which of the four search strategies participants used is that not a single person reported considering the object as a whole. We also talked to a few participants informally after the experiment and the post-experimental question. Most agreed that the search was very difficult; they considered the idea absurd that one could find the targets by looking for the object as a whole instead of focusing on one of its parts. Moreover, only four participants reported to have switched between the two object parts during search.

## Discussion

### Attention directs actions in visual foraging

The first notable finding of the present study is that the conjunction object stimulus worked to elicit run-like foraging behavior. This is evident from the below-chance-level object-part switching probability we found. With about 0.41 on average (but for some participants even around 0.25), the tendency to switch is higher than often found for regular conjunction foraging, which is around 0.1 to 0.2 on this metric (see Tünnermann, et al.’s^29^ estimate based on Kristjánsson et al.’s ^26^ data). Because in our paradigm the object parts are spatially adjacent, unlike in regular conjunction foraging, where the alternatives are spatially separated, noise in the execution of the collecting actions is likely to increase variability at the point where the stylus makes contact. Nevertheless, the pattern we observed is statistically robust and present in all participants. Therefore, it seems that constraints in representing or switching between search templates that require feature conjunctions indeed led foragers to adopt “good enough” guidance strategies, limiting their guiding template to a part of the object^39^. Importantly, this object-part bias translated—though mitigated through motor noise—to a motor bias in the exact tap position.

The tendency to forage in sequential runs observed in the present and many earlier studies^26–29^ can be interpreted as revealing an attentional effect on action selection, significantly narrowing potential action targets by filtering out items that do not match the currently prioritized feature combination. This perspective, along with the new finding that attention is reflected in the fine-grained tap positions can be placed within an overarching framework that integrates attentional and motor selection processes: Early in the dorsal stream, attention modulates an array of representations of competing potential actions^23,24^ targeting the objects in a foraging patch. Feature-based filtering as described above and other factors such as prioritization of near-by objects, further prune the pool of possible actions. Neurophysiological studies provide direct evidence of these early-stage representations in dorsal processing, typically in scenarios requiring choices between two competing action alternatives^50^. Many efficiently handled real-world tasks and foraging in particular confront organisms with a large number of action possibilities, so it seems plausible to assume that action planning and specification proceeds in a similar way with arbitration between multiple targets. Such situations also highlight the need for early prioritization, to prevent the system from specifying in too great detail action plans that never become realized. According to Cisek and colleagues^23,24^, downstream processes arbitrate among action targets as well when further specifying the actions, incorporating influences from object identification and cognitive decision-making. In visual foraging, factors such as item value could contribute to this^51,52^. In the present study, if the target template and spatial attention prioritize nearby targets effectively, decision-making demands are minimal, if not absent, as all targets share equal value and require no further identification. Thus, early attention-based modulation of competing action targets primarily drives action specification.

The tap positions in our study, which reflect the attended object part, reveal that this modulation is remarkably fine-grained, with action target locations specified at high resolution. Yet, tap distributions seem influenced by more than attention alone. For 16 out 20 participants, the center of the tap distribution was shifted into the rectangular object part. The reason for this might be that in parallel to the action specification via attention, motoric factors come into play. The center of mass has been shown to determine grasp contact points on objects^53–55^, which are automatically updated while the action is executed^56–58^. Even in our two-dimensional scenario the center of mass, which is located slightly off-center of the full object in the rectangular part, is a reliable stable contact point for the pen tip, and directing actions toward it could be a concurrent process of action target specification alongside attention. Similarly, action planning often prefers low precision requirements^59,60^, potentially steering actions toward the larger rectangular region. In sum, the observed tap distribution seems shaped by both attentional and motor factors, suggesting an interplay where attention provides fine-grained guidance, while motoric factors further refine action targeting.

That the observed repeated taps on one object part are likely a combination of attentional priority and such motor biases (and not driven by the motor biases alone) can be seen when relating the reported object-part preferences to the estimated observed tapping preferences. If the bias was purely motoric, it should not matter which object part the observers attended to. But as Fig. 6G shows, participants who reported having attended to the circular part were less likely to tap on the rectangular part. Nevertheless, they seemed to have been biased somewhat to the rectangular part, a data pattern one would expect when attentional and motoric biases work in opposite directions. Note that other motoric factors that might affect the contact point location, such as object orientation or location in the display, could not explain our findings, as these factors were randomized.

A question that may arise at this point is how our findings relate to similar influences on the control of eye movements. So far, no visual foraging experiments have examined whether fixations are biased toward attended object parts. However, in a different paradigm, Edelman and colleagues^61^ demonstrated that a visuomotor set, created by instructing participants to saccade to the left or right of a spatially adjacent pair of objects appearing simultaneously (or, in a similar experiment, to the left or right side of a single stimulus), biased express saccades toward the instructed side. With the adjacent stimulus pair, the saccade endpoint was only partially biased toward the instructed side—similar to how our data shows that contact points are only partially shifted toward the attended object part. While Edelman et al. employed an instructed visuomotor set (a relative spatial location) and studied express saccades toward abrupt onsets, our experiment investigated a feature-induced attention bias affecting manual reaches toward static targets. Despite these differences, both studies demonstrate that attentional or cognitive processes can directly bias motor responses. Future research should further explore how gaze and manual responses interact with attention biases in determining contact points in visual foraging.

In summary, our study reinforces the notion that attention and action are deeply interconnected. It highlights that attention not only selects between competing objects for action but also provides precise spatial information that fine-tunes goal-directed movements. However, selective attention is not the sole factor guiding these actions; motor influences also shape the final target locations. This agrees well with models that view perception and action not as separate, sequential stages, but as parallel streams that interact continuously during the processing of information^23,24^.

### Visual foraging as a task for investigating the interplay of attention and action

To conclude this article, we would like to take a step back and discuss why we consider visual foraging tasks a promising framework for assessing the interplay between selective attention and goal directed actions. Firstly, it possesses a naturalistic quality in many aspects that are pertinent to attention and action. For instance, the stimulation does not contain any abrupt onsets (except one when the patch is initially shown), masks, or other artificial elements that some experimental paradigms require. With regard to the response, the selection actions (especially when performed by finger or stylus touch) align more closely with natural behavior compared to button presses used in many experimental paradigms.

As suggested by Yu et al.^39^, selective attention frequently relies on “good enough” information, which can be swiftly processed, even if it occasionally leads to selection errors. This might be particularly true if the cost of errors is low. In many real-world foraging tasks, especially if the target features remain the same over a longer time, this is the case. In berry picking, for example, foragers might quickly select and pluck berries based on approximate criteria such as the color indicating ripeness. Such guidance is “good enough” because selection errors have low cost: An unripe berry can quickly be discarded or perhaps be eaten anyway, or the picked berries undergo a closer inspection in a later sorting phase. Consequently, absolute accuracy is not imperative at the moment of selection, and early attentional information potentially feeds into the motor system rather directly without incurring costly intermediary decision-making processes, as we have seen in the present study. Hence, the impact of attention might be more conspicuous in tasks like foraging compared to other visually guided behaviors where errors carry higher costs or decision processes cannot be deferred. On a side note, the cost of errors can be easily manipulated in visual foraging experiments. Not penalizing errors beyond the invested time and energy, or employing abstract (and technically inconsequential) minus-points as in the present study, results in low-cost errors. Freezing item collecting or terminating the trial upon selection of a distractor^26^ may be experienced as more costly errors. Other penalties, such as reducing monetary rewards, are likely perceived as even higher costs. The impact of selection error cost on the strength of attentional influences in foraging has not yet been investigated systematically, but it seems that manipulating error cost could be a promising avenue for testing the aforementioned rationale in future research.

Additionally, there are theoretical considerations that render the visual foraging tasks interesting for investigating attention and action. Recently, Schmalbrock et al.^62^ argued that the displays typically used in action-control research are too sparse to unveil the influence of salience on action control. When displays contain few items only, observers may resort to a search mode based on clump scanning^63^, in which a small set of stimuli can be processed in parallel. In such a case, the search is not driven by priority guidance and hence effects such as those exerted by stimulus salience may not manifest. Schmalbrock et al. addressed this by employing a circular arrangement of stimuli with a larger-than-usual number of items (8 letters), which they contrasted with a relatively smaller set (4 letters). Indeed, they only observed evidence for an impact of salience on stimulus–response bindings (a presumed joint representational format for action and perception^22^) with the larger display size. The visual foraging task seems to improve on this further. Patches contain many stimuli (84 in the experiment of the present study) which can densely populate the display area.

From a research-practical perspective, visual foraging tasks yield a substantial volume of data in a short span of time with little participant instruction or training. Typically, foragers execute multiple selection actions within a second. By contrast, traditional visual search experiments, particularly those involving actions beyond button pressing, require several seconds for a single trial to unfold. In sum, the visual foraging task is a highly promising paradigm to investigate attention and action from both practical and theoretical standpoints.

## Supplementary Information

Supplementary Information.

## Data Availability

The data and analyses scripts can be found at https://osf.io/cyrn6/.
