# Supplementary material for: Attention directs actions in visual foraging
Source: Sci Rep. 2025 May 6;15:15788. doi: 10.1038/s41598-025-97986-1 (PMC12056034; doi:10.1038/s41598-025-97986-1)
Supplement: Supplementary file 1 — Supplementary Information. [file 41598_2025_97986_MOESM1_ESM.pdf]

Supplementary Information for Attention Directs Actions in Visual Foraging

Jan Tünnermann and Anna Schubö

Cognitive Neuroscience of Perception and Action, Department of Psychology,

Philipps-University Marburg, Germany.

Switching Probability Model

The model used for estimating the switching probability is based on the hierarchical Bayesian beta-binomial model suggested by Albert and Hu[49] (Chapter 10) and is similar to the ones we used for other visual foraging data[29,30]. It provides group- and participant-level estimates of the probability with which participants switch between the object parts. If the estimate is (robustly) below .5, it means that the foragers produce same-part runs, at least to some degree, with greater length than what would be expected by mere chance repetitions of the same part. Note that unlike in foraging experiments where the options to switch or not are increasingly constrained due to the removal of certain item types from the display (where binomial models would not be applicable), foragers in our experiment always had all options, as clicking on either part could collect the conjunction objects.

The model structure and priors are described below and show how the parameters of interest are obtained from the binary switch/no-switch data y^{switch}:

$$
\begin{aligned}
p_\mu^{\text{switch}} &\sim \text{Beta}(1\,, 1) \\
\log \eta &\sim \text{Logistic}(\log 41\,, 1) \\
\eta &\leftarrow \exp(\log \eta)
\end{aligned}
$$

$$\alpha\,,\beta \quad \leftarrow \quad p_{\mu}^{\text{switch}} \cdot \eta\,,\left(1 - p_{\mu}^{\text{switch}}\right) \cdot \eta$$

$$p_i^{\text{switch}} \quad \sim \quad \text{Beta}(\alpha\,,\beta), \text{ for } i = 1, \ldots, |\text{Participants}|$$

$$y_i^{\text{switch}} \quad \sim \quad \text{Binomial}(41\,, p_i^{\text{switch}})$$

The Beta(1, 1) prior on p_{μ}^{switch}, the group-level estimate of the switching probability, reflects equal a priori probability for all values from 0 to 1. The Logistic prior on $\log \eta$ implements a flat prior on the degree of shrinkage for a representative number of 41 switch possibilities (see [49]). The implementation and estimation procedure can be found in *AnalyzeSwitchProb-AndPreference.ipynb* at https://osf.io/cyrn6/.

Preference Model

The preference model is similar to the switch model but does not include a group-level layer, as we did not have the assumption that there is common tendency in the group to prefer either of the object parts. The probability of preferring the red object part (an arbitrary choice) $p_i^{\text{preference}}$ is estimated from the observed choices $y^{\text{preference}}$ as follows:

$$p_i^{\text{preference}} \quad \sim \quad \text{Beta}(1\,,1), \text{ for } i = 1, \ldots, |\text{Participants}|$$

$$y_i^{\text{preference}} \quad \sim \quad \text{Binomial}(42\,,\; p_i^{\text{preference}})$$

The Beta(1, 1) prior assigns equal a priori probability to all values from 0 to 1. The implementation of this model and a very similar version that is applied to blocks of trials to produce the analysis of switching probability over time can be found in *AnalyzeSwitchProbAndPreference-.ipynb* at https://osf.io/cyrn6/.

Split-half Model

The split-half model was used to estimate the correlation between the switching probabilities from the first and second half of the experiment. The group level was modeled on the log-odds scale with a multivariate normal distribution with an LKJ prior for the Cholesky decomposition of the covariance matrix (as implemented in PyMC[44]), from which the correlation was obtained. On the participant level, a binomial model, as described above, was used.

$$\sigma_h \sim \text{Exponential}(1), \text{ for } h = 1, 2 \text{ (halves)}$$

$$\mu_h \sim \text{Normal}(0, 1.5), \text{ for } h = 1, 2$$

$$\text{chol, corr} \sim \text{LKJCholeskyCov}(\eta = 2, n = 2,\ \sigma = \sigma_h)$$

$$l_i^h \sim \text{MvNormal}(\mu_h,\ \text{chol})$$

$$p_i^h \leftarrow \text{invlogit}(l_i^h)$$

$$y_i^h \sim \text{Binomial}(42, p_i^h)$$

The priors are conservative: the prior on μ_h is centered at zero (which is .5 on the probability scale) and the $\eta = 2$ in LKJCholeskyCov also puts most prior density on weak correlations. As can be seen in the results (see main text), these priors were clearly overruled by the data. See *AnalyzeSwitchProbAndPreference.ipynb* at https://osf.io/cyrn6/ for further details.

Sampling Process

The models were implemented in PyMC[44]. We obtained 20,000 posterior samples from the models using the NUTS sampler[45], except for the simulation-based power analysis, where we limited sampling to 2,000 iterations. Further details on the sampling process can be found at https://osf.io/cyrn6/.

Simulation-based Power Analysis

While there is no notion of power as the reduction of the beta error probability in Bayesian statics in the traditional sense, it nevertheless seems reasonable to ensure power in the sense of increasing the chances of obtaining informative outcomes (see [43]). The general rationale of this approach is to repeatedly simulate experimental data, including all relevant assumptions, running the data through the same model that is planned for the actual experimental data, and estimating power as the proportion with which the research goals (e.g., finding the desired effect) are reached. This enables fine-grained power analyses that can include many parameters such as the number of trials, number of participants, etc. In the present study, the number of trials and the number of targets, which affect power, are fixed based on practical decisions (e.g., targeted overall duration of the experiment, desired stimulus density in the display, etc.). Consequently, only the number of participants can be scaled to adjust the power. To this end, we simulated 100 replications for 4 to 30 participants (in steps of two) and calculated the power of observing sub-random switching that indicates run-like clicking on the object parts. We consider four different success criteria: switching probability with upper HDI boundary below .5, and below values of .49, .48, and .47. While the .5 criterion already signals statistically robust sub-random switching, the other variants include larger separations to the critical value, more narrowly encompassing the actual switching probability. Table 1 lists all the chosen fixed parameter values and assumptions for the simulation, and briefly states why we believe these are reasonable and conservative choices. Some values are informed by earlier foraging experiments, while others include insights obtained from three pilot datasets (see *PowerAnalysis.ipynb* at https://osf.io/cyrn6/).

Table A1

Parameter choices

Parameter	Value	Motivation/Justification
**trials**	20	Fixed; enables data recording in about 30 min; typical value (e.g., Á. Kristjánsson et al.[26] used 20 trials per condition).
**targets**	42	Fixed; fills, together with an equal number of distractors, the 12 × 7 grid of a patch (close to Kristjánsson et al.'s[26] 40 targets per patch).
**deflection**	3.5 pix.	This is assuming a very small deflection away from the object center toward the attended part of 3.5 pixels on average. The absolute deviation from the object center we observed in three pilot datasets was 3.79 pixels (see *PowerAnalysis.ipynb*). Note that this strongly underestimates the true deflection toward the attended part, as it ignores which part is attended when. For instance, when there is a high deflection of 10 pixels, but the participant attends to one of the parts in half of the trials and to the other part in the other half, the deflection averages out to 0. Such canceling out is expected, at least to some degree, and hence, this assumption is quite conservative.
**sigma**	9	Sigma is the standard deviation of the click position modeling motor noise. For a rough reference, we looked at the average standard deviation of the clicks in our three pilot datasets along the axis that is orthogonal to the one of interest (sigma = 8.25, see *PowerAnalysis.ipynb*). That is, after normalizing all clicks into upright target orientation along the y-axis (so that one object part is above the other), we looked at the standard deviation in the x-dimension. This dimension is unaffected by the up/down object part choice and, consequently, a better reflection of the motor noise compared to the y-dimension, where some dispersion comes from targeting the upper and lower parts. However, as participants could click the object in its full width (twice as wide as the parts are tall), the precision requirements were substantially lower. Assuming that participants are more variable when not required to be precise, this reflects a conservative choice for this parameter.
**sw_prob**	.1	Assumed attentional template switching probability. We averaged the observed switches from Kristjánsson et al.'s[26] data (excluding super-foragers) to obtain a rough estimate of the expected switching probability when foraging for conjunctions (sw_prob = .09, see *PowerAnalysis.ipynb*). Note that in Kristjánsson et al.'s [26], participants had to switch at least once, which is not true for the present study, where participants can stick to one target for the whole trial. Hence, template switching might be even less probable in our scenario, which renders 0.1 a conservative choice.
**sw_prob_sf**	.5	Assumed switching probability of super-foragers, a subset of participants that switches flexibly also in conjunction foraging (see Kristjánsson et al.[26]). Estimated from Kristjánsson et al.'s[26] data, as described above for sw_prob, leads to a value of sw_prob_sf = .35 (see *PowerAnalysis.ipynb*). Higher values work against the hypothesized effect, making .5 a very conservative choice.
**sf_prop**	.25	The proportion of super-foragers in a typical sample is around 25 %; see Kristjánsson et al.[26] and Jóhannesson et al.[35].

Algorithm 1 Simulate one experiment

```
function SIMULATE(participants, trials, targets, deflection, sigma,
                  switch_prob, switch_prob_sf, sf_prop)
dataframe ← initialize as empty
for participant in (1 to participants) do
   for trial in (1 to trials) do
      attended_part ← 1
      picked_parts ← [ ]
      for target in (1 to targets) do
         if participant < participants · sf_prop then
            switch = Bernoulli(switch_prob_sf) {for the super foragers}
         else
            switch = Bernoulli(switch_prob) {for others}
         end if
         if switch = 1 then
            attended_part = attended_part · −1 switch between parts
         end if
         picked_pos ← Normal(attended_part, sigma) {click with noise}
         if picked_pos <= 0 then
            append "lower" to picked_parts
         else
            append "upper" to picked_parts
         end if
         switches ← where picked_parts changes between "lower" and "upper"
         repetitions ← length of switches vector
         append switches and repetitions to for this trial of this participant
         to dataframe
      end for
   end for
end for
return dataframe
end function
```

Note. Algorithm that simulates a dataset of the experiment with a certain number of participants. It is repeatedly called by the power analysis script (Algorithm 2). The Python implementation can be found in *simulator.py* at https://osf.io/cyrn6/.

Algorithm 2 repeatedly calls the simulation for different participant counts, fits the data with the model (see section "Switching probability model"), records whether the effect was found, and, in the end, calculates the power estimate and its 95% HDIs. In the actual implementation (see *PowerAnalysis.ipynb* at https://osf.io/cyrn6/), the estimates are saved into files, which are then

read for plotting the development of power over the participant count (see *PlotPower.ipynb* at https://osf.io/cyrn6/). The resulting chart can be seen in Figure A1.

Algorithm 2 Power simulations

```
{Parameters:}
replications ← 100
participant_counts ← [4, 6, 8, 10, 12, 14, 16, 18, 20, 22, 24, 26, 28, 30]
trials ← 20
targets ← 42
deflection ← 3.5
sigma ← 9
switch_prob ← 0.1
switch_prob_sf ← 0.5
sf_prop ← 0.25

{Run simulations}
success_dataframe ← empty
for each participant_count in participant_counts do
  for each replication in (1 to replications) do
    simulate_dataset ← SIMULATE(p, trials, targets, deflection, sigma,
                        switch_prob, switch_prob_sf, sf_prop)
    model ← construct model for simulate_dataset
    trace ← model.sample(2000 samples)
    success_at_50 ← trace.switching_probability.upper_HDI_bound < 0.50
    success_at_49 ← trace.switching_probability.upper_HDI_bound < 0.49
    success_at_48 ← trace.switching_probability.upper_HDI_bound < 0.48
    success_at_47 ← trace.switching_probability.upper_HDI_bound < 0.47
    append success_at_50, success_at_49, success_at_48, and success_at_47 to
    success_dataframe
  end for
end for

{Calculate power}
power_point_estimates ← mean success from success_dataframe
power_HDIs ← HDIs calculated via beta distribution
```

Note. Algorithm for the power analysis. The Python implementation can be found as *PowerAnalysis.ipynb* at https://osf.io/cyrn6/.

As can be seen in Figure A1, the expected power for detecting sub-random switching with twelve participants is already high at .97 [.92, .99]$^{HDI95\%}$. At twenty participants, the power for detecting sub-random switching approaches 1 [.97, 1]$^{HDI95\%}$, Moreover, at twenty, the power for finding sub-random switching with an upper HDI boundary below .49 has also risen to a substantial value of .91 [.84, .96]$^{HDI95\%}$. It can also be seen in the figure that further increasing the participant count

does not substantially increase the chances of further narrowing the HDI of the switching probability so that the upper boundary would be below .48 or .47. Hence, recording twenty participants seems a reasonable choice.

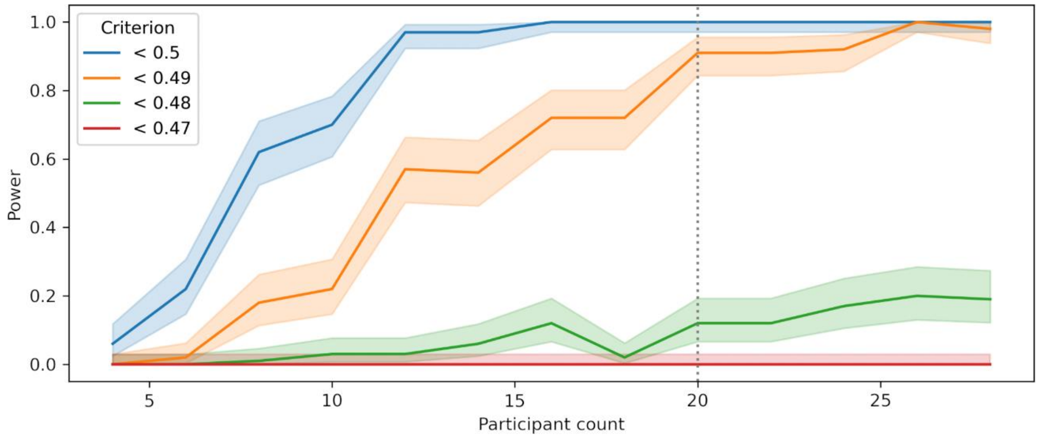

Figure A1. Results of the power analysis. Development of power with increasing participant count for four different success criteria. The dotted line indicates the selected participant count of twenty.

References

See main text.
